# Contour Detection and Completion for Inpainting and Segmentation Based on Topological Gradient and Fast Marching Algorithms

**DOI:** 10.1155/2011/592924

**Published:** 2011-12-11

**Authors:** Didier Auroux, Laurent D. Cohen, Mohamed Masmoudi

**Affiliations:** ^1^Laboratoire J. A. Dieudonné, Université de Nice Sophia Antipolis, Parc Valrose, 06108 Nice Cedex 2, France; ^2^CEREMADE, UMR CNRS 7534, Université Paris Dauphine, Place du Marchal De Lattre De Tassigny, 75775 Paris Cedex 16, France; ^3^Institut de Mathématiques de Toulouse, Université Paul Sabatier, 31062 Toulouse Cedex 9, France

## Abstract

We combine in this paper the topological gradient, which is a powerful method for edge detection in image processing, and a variant of the minimal path method in order to find connected contours. The topological gradient provides a more global analysis of the image than the standard gradient and identifies the main edges of an image. Several image processing problems (e.g., inpainting and segmentation) require continuous contours. For this purpose, we consider the fast marching algorithm in order to find minimal paths in the topological gradient image. This coupled algorithm quickly provides accurate and connected contours. We present then two numerical applications, to image inpainting and segmentation, of this hybrid algorithm.

## 1. Introduction

Contour detection is a major issue in image processing. For instance, in classification and segmentation, the goal is to split the image into several parts. This problem is strongly related to the detection of the connected contours separating these parts. It is quite easy to detect edges using local image analysis techniques, but the detection of continuous contours is more complicated and needs a global analysis of the image.

Several image processing problems like image inpainting and denoising (or enhancement) are classically solved without detecting edges and contours. The goal of image enhancement is to denoise the image without blurring it. A classical idea is to identify the edges in order to preserve them and to smooth the image outside them. In this particular case, contour completion is not prerequisite, as the quality of the result is not too much related to the completeness of the identified edges, but missing edges may lead to blurred boundaries. For most of the other image processing problems (segmentation, inpainting, classification), the detection of connected contours can drastically simplify the resolution and improve the quality of the results. For instance, the image segmentation problem is a very good example, as the goal is to split the image into its characteristic parts. In other words, one has to find connected contours, which define different subsets of the image.

For solving all these problems, various approaches have been considered in the literature. We can cite here the most commonly used models: the structural approach by region growing [1], the stochastic approaches [2–4], and the variational approaches, which are based on various strategies like level set formulations, minimizing the total variation of a quantity or the Mumford-Shah functional, active contours and geodesic active contours methods, snakes, wavelet transforms, or shape gradient [5–19, 19–24].

Another approach is based on the topological asymptotic analysis and consists of defining edges as cracks [25, 26]. The goal of topological optimization is to look for an optimal design (i.e., a subset) and its complementary. Finding the optimal subdomain is equivalent to identifying its characteristic function. At first sight, this problem is not differentiable. But the topological asymptotic expansion gives the variation of a cost function *j*(*Ω*) (see Section 2 for examples) when one switches the characteristic function from one to zero (or from zero to one) in a small region [27].

More precisely, we consider the perturbation of the main domain *Ω* by the insertion of a small crack (or hole) *σ*_*ρ*_ : *Ω*_*ρ*_ = *Ω*∖*σ*_*ρ*_, *ρ* being the size of the crack. The topological sensitivity theory provides then an asymptotic expansion of the considered cost function when the size of the crack tends to zero. It takes the general form: *j*(*Ω*_*ρ*_) − *j*(*Ω*) = *f*(*ρ*)*g*(*x*) + *o*(*f*(*ρ*)), where *f*(*ρ*) is an explicit positive function going to zero with *ρ*, and *g*(*x*) is the topological gradient at point *x*. Then, in order to minimize the criterion (or at least its first order expansion), one has to insert small cracks at points where the topological gradient is the most negative. Using this gradient type information, it is possible to build fast algorithms. In most applications, a satisfying approximation of the optimal solution is reached at the first iteration of the optimization process. A topological sensitivity framework allowing to obtain such an expansion for general cost functions has been proposed in [27].

An efficient edge detection technique, based on the topological gradient, has been introduced in [28, 29]. It is also shown that edge detection can make all these image processing problems straightforward to solve [25, 26, 30, 31]. But the identified edges are usually not connected, and the results can be degraded. Our goal is to improve these results by replacing dashed discontinuous edges by connected contours.

In the inpainting problem, we assume that there is a hidden part of the image, and our goal is to recover this part from the known part of the image. We assume that the missing part is a quite large part of the image, we do not consider the case of random sets or narrow lines. This problem has been widely studied and the most common approaches are: learning approches (neural networks, radial basis functions,…) [32, 33], minimization of an energy cost function based on a total variation norm [34, 35], morphological component analysis methods separating texture and cartoon [36]. We also refer to [6, 8] for the description of several inpainting algorithms.

We now consider the crack detection technique, within the framework of the identification of the image edges, either in the hidden part of the image for the inpainting application, or in the whole image for the segmentation application [26]. The topological asymptotic analysis provides very quickly the location of the edges, as they are precisely defined by the most negative points of the topological gradient. The great advantage of the topological gradient in comparison with level line completion and TV-based inpainting methods (see e.g., [6, 8, 12, 13]) is that the identified edges in the unknown part of the image correspond to a regular extrapolation of the known edges, and as we will see on a numerical example, the topological gradient preserves the continuity of the edge curvature. Thus, the proposed approach is much more than simple edge detection.

The main issue of the approach based on the topological gradient is the need for connected complete contours. This can be easily understood since the hidden part of the image is filled in using the Laplace operator in each subdomain of the missing zone, and a discontinuous contour would lead to some blurred reconstruction. Up to now, one had to threshold the topological gradient with a not too small value, in order to identify connected contours, but this leads to thick identified edges, and also to consider more noisy points as potential edges. In order to overcome this limitation, we consider a minimal path technique in order to connect the edges identified by the topological gradient.

Minimal paths have been first introduced for finding the global minimum of active contour models, using the fast marching technique [37, 38]. They have then been used to find contours or tubular structures and also for perceptual grouping using a path or a set of paths minimizing a functional [38–43]. In our case, the energy to be minimized will be an increasing function of the topological gradient. As the topological gradient takes its minimal (negative) values on the edges of the image, the idea is indeed to find contours for contour completion from the various minima and small values of the topological gradient.

The energy to be minimized can be seen as a distance function. The idea is then to compute this distance function between a given starting point and all other points. For this purpose, a front propagation equation is considered. Using the fast marching propagation, the definition of the distance function is straightforward: the distance between a point *x* and the starting point is exactly the time at which the front reached *x*. Then, minimal paths between these points can be identified using a gradient descent. For perceptual grouping, a set of keypoints is considered as starting points and a set of minimal paths connecting some pairs of these keypoints is considered as a contour completion. This approach is extremely satisfactory in 2D problems, with quite few key points. It is also extremely fast. In 3D images, minimal paths find tubular structures, but in order to identify minimal surfaces, this approach is much more difficult to consider. It was dealt in the case of a surface connecting two curves in [44]. We only consider here the 2D case.

The application of the minimal path technique to the topological gradient allows us to obtain an automatic identification of the main (missing or not) edges of the image. These edges will be continuous, by construction, and will allow us to simply apply the Laplace operator to fill in the image for inpainting applications, or will directly provide the segmented image, with very good results. Another advantage of this technique is to be very fast, as it does not degrade the *𝒪*(*n* · log⁡(*n*)) complexity of the topological gradient based algorithm introduced in [26]. We refer to [26, 45] for the inpainting and segmentation algorithms by topological asymptotic expansion, and for a detailed presentation of the topological gradient.

The paper is organized as follows. In Section 2, we present the edge detection method using the topological gradient, and the corresponding segmentation and inpainting algorithms. In Section 3, we propose an algorithm based on the minimal path and fast marching techniques in order to identify the valley lines of the topological gradient, which correspond to the main edges of the image. Then, we report the results of several numerical experiments in Section 4. We also compare this hybrid scheme with the fast marching algorithm applied to the standard gradient. Two particular image processing problems are considered: segmentation and inpainting. Finally, some conclusions are given in Section 5.

## 2. Edge Detection by Topological Asymptotic Analysis and Its Application to Inpainting and Segmentation

### 2.1. Topological Asymptotic Analysis

Let *Ω* be an open bounded domain of ℝ^2^ (note that it can easily be extended to ℝ^*n*^). We consider a partial differential equation (PDE) problem defined in *Ω*, and we denote by *u*_*Ω*_ its solution (we will further see under which assumptions it can be considered). We finally consider a cost function *J*(*Ω*, *u*_*Ω*_) to be minimized, where *u*_*Ω*_ is the solution to the PDE in *Ω*. The idea of topological asymptotic analysis is to measure the impact of a perturbation of the domain *Ω* on the cost function.

**Figure 1:**
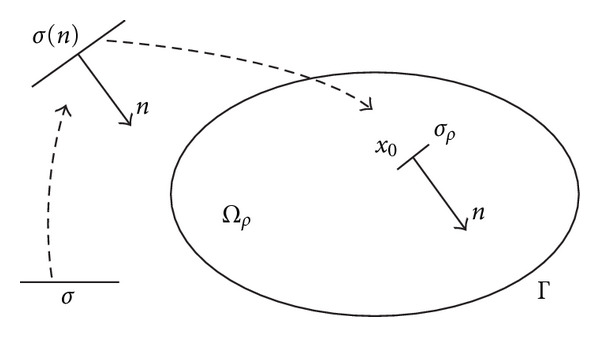
Example of domain and inserted crack (with its orientation).

For a small *ρ* ≥ 0, let *Ω*_*ρ*_ = *Ω*∖*σ*_*ρ*_ be the perturbed domain by the insertion of a crack *σ*_*ρ*_ = *x*_0_ + *ρσ*(*n*), where *x*_0_ ∈ *Ω*. We denote by *σ* a fixed bounded straight crack containing the origin, *n* is a unit vector, and *σ*(*n*) is the result of the rotation of *σ* so that *n* is the normal to *σ*(*n*). The fixed crack *σ* is rotated (normal *n*), stretched (size *ρ*), and translated (center *x*_0_) in order to get *σ*_*ρ*_ (see Figure 1). The topological gradient theory can also be applied in the case of arbitrary shaped holes [46–49], but we will only consider the case of crack perturbations in our applications. The small parameter *ρ* will represent the size of the inserted crack. Finally, we denote by *𝒱* a Hilbert space on *Ω*, usually *H*^1^(*Ω*) in our applications.

We now consider the variational formulation of the PDE problem on *Ω*

(1) $$\begin{array}{c} \text{Find}\;u\in 𝒱\;\text{such}\;\text{that}\\ a\left(u,w\right)=l\left(w\right),\;\forall w\in 𝒱, \end{array}$$

and the corresponding variational formulation of the PDE problem on the perturbed domain

(2) $$\begin{array}{c} \text{Find}\;u_{\rho }\in {𝒱}_{\rho }\;\text{such}\;\text{that}\\ a_{\rho }\left(u_{\rho },w\right)=l_{\rho }\left(w\right),\;\forall w\in {𝒱}_{\rho }. \end{array}$$

One should notice that for *ρ* = 0, the perturbed PDE problem becomes the original PDE problem.

We assume in the following that *a*_*ρ*_ is a bilinear continuous and coercive form defined on *𝒱*_*ρ*_, a Hilbert space on *Ω*_*ρ*_, and that *l*_*ρ*_ is a linear continuous form on *𝒱*_*ρ*_.

We can rewrite the cost function *J* as a function of *ρ* by considering the following map:

(3) $$\begin{array}{cc} & j:\rho \mapsto \Omega_{\rho }\mapsto u_{\rho },\;\text{solution}\;\text{of}\;\text{Equation}\;\left(2\right)\mapsto j\left(\rho \right)\\ & \;\;∶=J\left(\Omega_{\rho },u_{\rho }\right). \end{array}$$

In order to apply the topological asymptotic theory, *a*_*ρ*_, *l*_*ρ*_, and *J* have to satisfy the hypotheses of the following result [50, 51].

If there exist a linear form *L*_*ρ*_ defined on *𝒱*_*ρ*_, a function *f* : ℝ^+^ → ℝ^+^, and four real numbers *δJ*_1_, *δJ*_2_, *δa*, and *δl* such that

(1)  lim⁡_*ρ*→0_*f*(*ρ*) = 0,
(2) *J*(*Ω*_*ρ*_, *u*_*ρ*_) − *J*(*Ω*_*ρ*_, *u*_0_) = *L*_*ρ*_(*u*_*ρ*_ − *u*_0_) + *f*(*ρ*)*δJ*_1_ + *o*(*f*(*ρ*)),
(3) *J*(*Ω*_*ρ*_, *u*_0_) − *J*(*Ω*, *u*_0_) = *f*(*ρ*)*δJ*_2_ + *o*(*f*(*ρ*)),
(4) (*a*_*ρ*_ − *a*_0_)(*u*_0_, *p*_*ρ*_) = *f*(*ρ*)*δa* + *o*(*f*(*ρ*)),
(5) (*l*_*ρ*_ − *l*_0_)(*p*_*ρ*_) = *f*(*ρ*)*δl* + *o*(*f*(*ρ*)),

where the adjoint state *p*_*ρ*_ is solution of the adjoint equation

(4) $$\begin{array}{c} a_{\rho }\left(w,p_{\rho }\right)=-L_{\rho }\left(w\right)\;\forall w\in {𝒱}_{\rho }, \end{array}$$

and *u*_*ρ*_ is solution of the direct (2), then the cost function has the following asymptotic expansion:

(5) $$\begin{array}{c} j\left(\rho \right)-j\left(0\right)=f\left(\rho \right)g\left(x\right)+o\left(f\left(\rho \right)\right), \end{array}$$

where *g*(*x*) is the topological gradient, given by

(6) $$\begin{array}{c} g\left(x\right)=\delta J_{1}+\delta J_{2}+\delta a-\delta l. \end{array}$$

Indeed, from second and third items, *j*(*ρ*) − *j*(0) = *J*(*Ω*_*ρ*_, *u*_*ρ*_) − *J*(*Ω*, *u*_0_) = *L*_*ρ*_(*u*_*ρ*_ − *u*_0_) + *f*(*ρ*)(*δJ*_1_ + *δJ*_2_) + *o*(*f*(*ρ*)). From the definition of the adjoint state and the direct equation, *L*_*ρ*_(*u*_*ρ*_ − *u*_0_) = −*a*_*ρ*_(*u*_*ρ*_, *p*_*ρ*_) + *a*_*ρ*_(*u*_0_, *p*_*ρ*_). From fourth item and direction (2), −*a*_*ρ*_(*u*_*ρ*_, *p*_*ρ*_) + *a*_*ρ*_(*u*_0_, *p*_*ρ*_) = −*l*_*ρ*_(*p*_*ρ*_) + *a*_0_(*u*_0_, *p*_*ρ*_) + *f*(*ρ*)*δa* + *o*(*f*(*ρ*)) = −*l*_*ρ*_(*p*_*ρ*_) + *l*_0_(*p*_*ρ*_) + *f*(*ρ*)*δa* + *o*(*f*(*ρ*)). Finally, from fifth item, this term is equal to *f*(*ρ*)(*δa* − *δl*) + *o*(*f*(*ρ*)).

Then, from an asymptotic point of view, as *f*(*ρ*) ≥ 0, the idea is to create cracks in the domain *Ω*, where the topological gradient *g* is the most negative, because

(7) $$\begin{array}{c} J\left(\Omega_{\rho },u_{\rho }\right)=J\left(\Omega ,u\right)+f\left(\rho \right)g\left(x\right)+o\left(f\left(\rho \right)\right), \end{array}$$

and the cost function corresponding to the perturbed problem will be smaller than the original one. The main advantage of this method is that it only requires the resolution of the direct (2) and adjoint (4) problems.

### 2.2. Application to Edge Detection

Let *Ω* be an open bounded domain of ℝ^2^, representing the image domain. For a given function *v* in *L*^2^(*Ω*) (in our application, *v* represents the input image), the initial problem is defined on the unperturbed domain and reads as follows: find *u* ∈ *H*^1^(*Ω*) such that

(8) $$\begin{array}{c} -\mathrm{div}\left(c\nabla u\right)+u=v\;\text{in}\;\Omega ,\\ \partial_{n}u=0\;\mathrm{on}\;\partial \Omega , \end{array}$$

where *n* denotes the outward unit normal to ∂*Ω* and *c* is a given function. Note that this problem is equivalent to linear diffusion restoration.

For a given *x*_0_ ∈ *Ω* and a small *ρ* ≥ 0, let us now consider *Ω*_*ρ*_ = *Ω*∖*σ*_*ρ*_ the perturbed domain by the insertion of a crack *σ*_*ρ*_ = *x*_0_ + *ρσ*(*n*), where *x*_0_ ∈ *Ω*, *σ*(*n*) is a straight crack, and *n* a unit vector normal to the crack. Then, the new solution *u*_*ρ*_ ∈ *H*^1^(*Ω*_*ρ*_) satisfies

(9) $$\begin{array}{c} -\mathrm{div}\left(c\nabla u_{\rho }\right)+u_{\rho }=v\;\text{in}\;\Omega_{\rho },\\ \partial_{n}u_{\rho }=0\;\mathrm{on}\;\partial \Omega_{\rho }. \end{array}$$

Edge detection is equivalent to looking for a subdomain of *Ω* in which the energy is small. Indeed, we consider the image gradient energy function, and the edges correspond to high variations of the image intensity, and then to high values of the gradient. So, our goal is to find the most energetic parts of the image (in order to identify the edges), and we reformulate this problem as the minimization of the energy norm outside the edges

(10) $$\begin{array}{c} j\left(\rho \right)=J\left(\Omega_{\rho },u_{\rho }\right)=\overset{}{\underset{\Omega_{\rho }}{\int }}{||\nabla u_{\rho }||}^{2}. \end{array}$$

Then, the cost function *j* has the following asymptotic expansion (see, e.g., [52] for more details):

(11) $$\begin{array}{c} j\left(\rho \right)-j\left(0\right)=\rho^{2}G\left(x_{0},n\right)+o\left(\rho^{2}\right), \end{array}$$

with

(12) $$\begin{array}{cc} G\left(x_{0},n\right) & =-\pi c\left(\nabla u_{0}\left(x_{0}\right)\cdot n\right)\left(\nabla p_{0}\left(x_{0}\right)\cdot n\right)\\ & \;-\pi {\left|\nabla u_{0}\left(x_{0}\right)\cdot n\right|}^{2}, \end{array}$$

and where *p*_0_ is the solution to the adjoint problem

(13) $$\begin{array}{c} -\mathrm{div}\left(c\nabla p_{0}\right)+p_{0}=-\partial_{u}J\left(\Omega ,u_{0}\right)\;\text{in}\;\Omega ,\\ \partial_{n}p_{0}=0\;\mathrm{on}\;\partial \Omega . \end{array}$$

The topological gradient could be written as

(14) $$\begin{array}{c} G\left(x,n\right)=\left(M\left(x\right)n\right)\cdot n, \end{array}$$

where *M*(*x*) is the 2 × 2 symmetric matrix defined by

(15) $$\begin{array}{cc} M\left(x\right) & =-\pi c\frac{\nabla u_{0}\left(x\right)\nabla p_{0}{\left(x\right)}^{T}+\nabla p_{0}\left(x\right)\nabla u_{0}{\left(x\right)}^{T}}{2}\\ & \;-\pi \nabla u_{0}\left(x\right)\nabla u_{0}{\left(x\right)}^{T}. \end{array}$$

For a given *x*, *G*(*x*, *n*) takes its minimal value when *n* is the eigenvector associated to the lowest eigenvalue *λ*_min⁡_ of *M*. This value will be considered as the topological gradient associated to the optimal orientation of the crack *σ*_*ρ*_(*n*).

Then, we can define the identified edge set

(16) $$\begin{array}{c} \sigma =\left\{x\in \Omega ;\lambda_{\min}\left(x\right)<\delta <0\right\}, \end{array}$$

where *δ* is a negative threshold.

**Figure 2:**
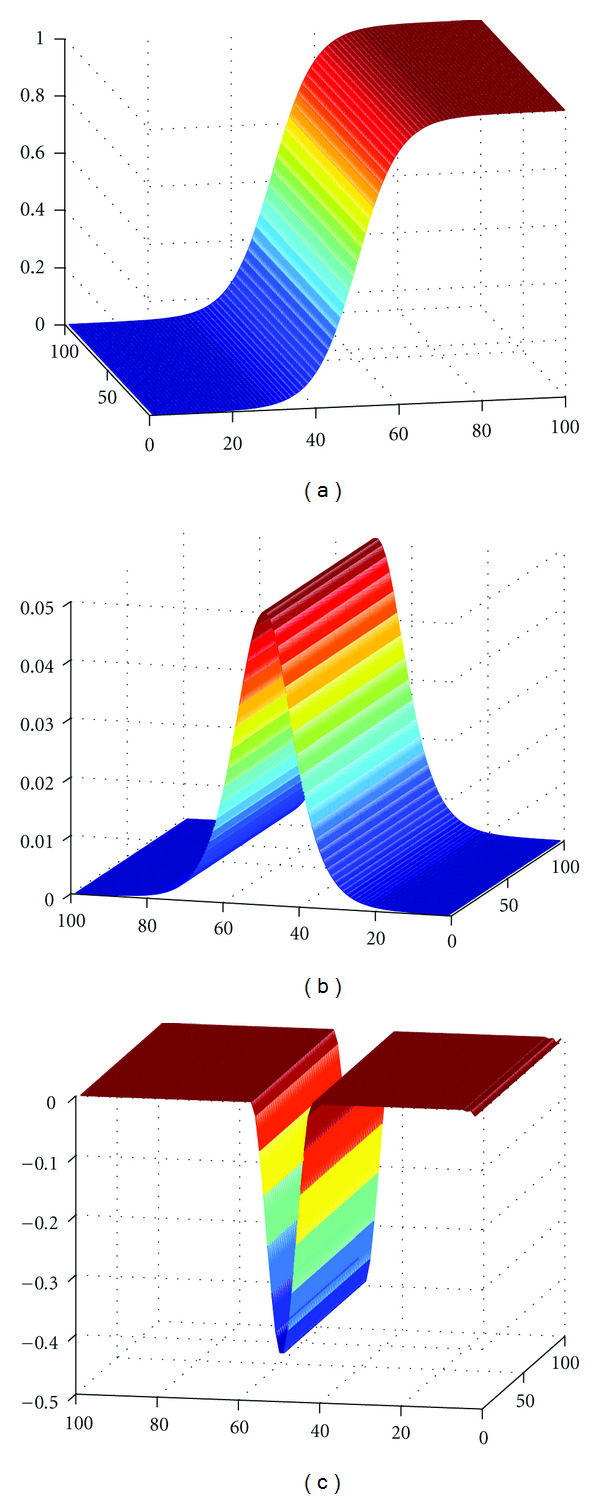
(a) Original image; (b) *L*^2^ norm of the (standard) gradient of (a); (c) Topological gradient of (a).

We first illustrate this technique on a synthetic two dimensional image, in grey level, defined by a sigmoid function in x-coordinate (cumulative distribution function of a Gaussian). The image is represented in Figure 2(a). Then, the *L*^2^ norm of its standard gradient ||∇*u*(*x*)|| and its topological gradient *λ*_min⁡_(*M*(*x*)) are represented in Figures 2(b) and 2(c), respectively.

One can see that the topological gradient is less sensitive to a smooth variation of the image intensity than the standard gradient. The support of the topological gradient is indeed much smaller. Thanks to the homogeneous Neumann condition on the crack, the solution of the perturbed problem is discontinuous along the crack, and the solution has a much smaller energy if one inserts a crack in the image near the middle of the *x*-axis.

**Figure 3:**
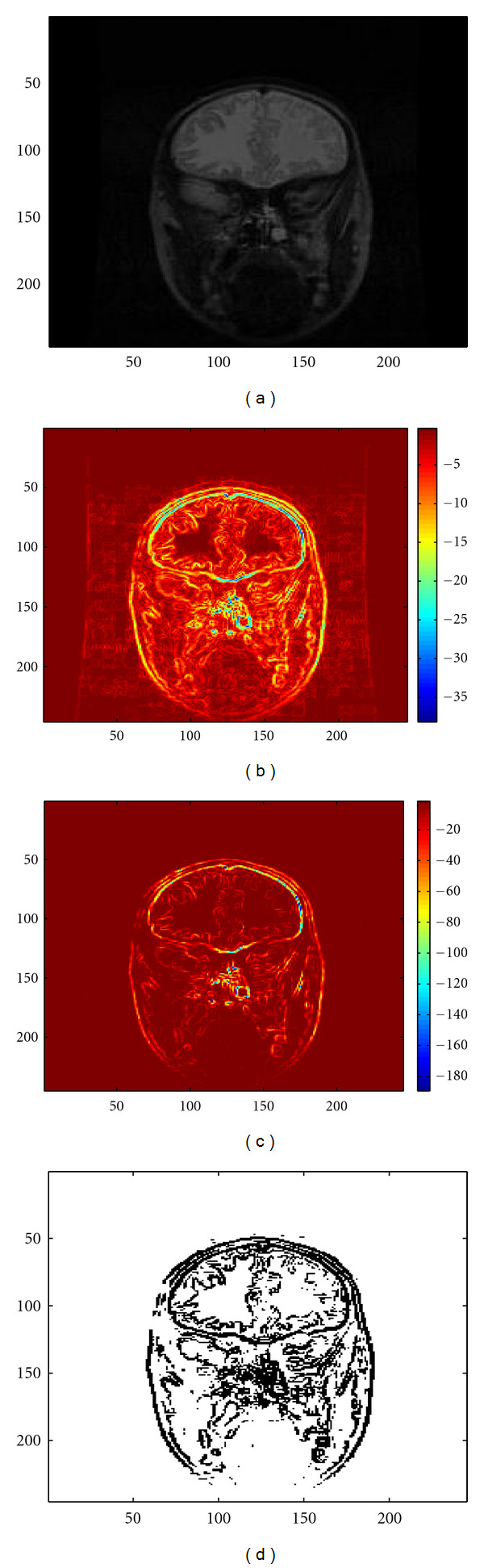
(a) Original image; (b) *L*^2^ norm of the (standard) gradient of (a); (c) Topological gradient of (a); (d) Identified edges by thresholding the topological gradient.

We now apply this edge detection technique to the image represented in Figure 3(a). The opposite of the *L*^2^ norm of its standard gradient is represented in Figure 3(b). Note that we represent its opposite in order to have comparable images with the topological gradient, which has negative values.

The topological gradient is represented on Figure 3(c). As it quantifies in a global way whether a pixel is part of an edge or not, it is much less sensitive to noise and small variations of the image than the standard gradient. For instance, the topological gradient takes much larger absolute values on the edges than outside, contrary to the standard gradient. Note also that the time required for the computation of the topological gradient is not much higher than for the standard gradient, thanks to the *𝒪*(*n* · log⁡*n*) complexity of the topological gradient algorithm.

However, for segmentation (or simply edge detection), the next step of topological gradient algorithms usually consists of thresholding the topological gradient in order to define the edge set. Such a threshold is represented in Figure 3(d). One can see that in order to obtain at least the main connected edge, the threshold coefficient has been set to a large value, leading to add many unwanted points to the edge set, but also to thick edges. And even in this case, the main contour is not totally continuous. This is why we need to hybridize this method with the fast marching algorithm (see Section 3.4) in order to obtain continuous edges for the segmentation and to remove the isolated unwanted pixels.

We will also see below that the fast marching algorithm needs a potential function highly related to the edges of the image, much more than the standard gradient of the image. Then, we will see that the topological gradient also improves the fast marching method within the segmentation framework, as the quality of the segmentation is directly related to the choice of the potential function.

### 2.3. Inpainting Algorithm by Topological Asymptotic Analysis

We also consider the inpainting application. We present here the topological gradient-based algorithm. Let *ω* ⊂ *Ω* be the missing part of the image and *γ* its boundary. We still denote by *v* the input image (assumed to be known in *Ω*∖*ω*, and unknown in *ω*). The algorithm is based on the fact that two measurements are available on the boundary of the hidden part of the image: the value of the image (Dirichlet condition) and its normal derivative (Neumann condition). From these two measurements, by considering the standard crack localization problem (see, e.g., [50]), it is possible to solve a Dirichlet problem and a Neumann problem for a given crack *σ*

(17) $$\begin{array}{c} \Delta u_{D}=0\;\text{in}\;\omega \setminus \sigma ,\\ u_{D}=v\;\mathrm{on}\;\gamma ,\\ \partial_{n}u_{D}=0\;\mathrm{on}\;\sigma ,\\ u_{D}=v\;\text{in}\;\Omega \setminus \omega , \end{array}$$

where *u*_*D*_ ∈ *H*^1^(*Ω*∖*σ*), and

(18) $$\begin{array}{c} \Delta u_{N}=0\;\text{in}\;\omega \setminus \sigma ,\\ \partial_{n}u_{N}=\partial_{n}v\;\mathrm{on}\;\gamma ,\\ \partial_{n}u_{N}=0\;\mathrm{on}\;\sigma ,\\ u_{N}=v\;\text{in}\;\Omega \setminus \omega , \end{array}$$

where *u*_*N*_ is in *H*^1^(*Ω*∖*σ*).

Then, in order to identify the missing edges, one has to minimize the following cost function:

(19) $$\begin{array}{c} J\left(\sigma \right)=\frac{1}{2}{||u_{D}-u_{N}||}_{L^{2}(\Omega )}^{2}. \end{array}$$

For the actual cracks (hidden edges), the solutions *u*_*D*_ and *u*_*N*_ should be equal, as the actual solution satisfies both Neumann and Dirichlet conditions. By minimizing this cost function, one tries to find a solution that is consistent with both conditions on the boundary.

The topological gradient corresponding to this cost function is given by

(20) $$\begin{array}{cc} G\left(x,n\right) & =-\;[\left(\nabla u_{D}\left(x\right)\cdot n\right)\left(\nabla p_{D}\left(x\right)\cdot n\right)\\ & \;\;\;+\left(\nabla u_{N}\left(x\right)\cdot n\right)\left(\nabla p_{N}\left(x\right)\cdot n\right)], \end{array}$$

where *p*_*N*_ and *p*_*D*_ are the two corresponding adjoint states [25, 50]. As previously, the topological gradient can be rewritten as *G*(*x*, *n*) = *n*^*T*^*M*(*x*)*n*, where *M*(*x*) is a symmetric matrix, and *G* takes its minimal value when *n* is the eigenvector associated to the lowest eigenvalue of *M*.

The inpainting algorithm is then the following:

- calculation of *u*_*D*_ and *u*_*N*_,
- calculation of *p*_*D*_ and *p*_*N*_,
- computation of matrix *M*(*x*) and its lowest eigenvalue *λ*_min⁡_ at each point of the missing domain *ω*,
- definition of the set of cracks: {*x* ∈ *ω*; *λ*_min⁡_(*x*) < *δ* < 0}, where *δ* is a negative threshold,
- dalculation of *u* solution to the Neumann problem taking into account the cracks location.

This algorithm has a complexity of *𝒪*(*n* · log⁡(*n*)), where *n* is the size of the image (i.e., number of pixels). We refer to [25] for more details about this algorithm.

**Figure 4:**
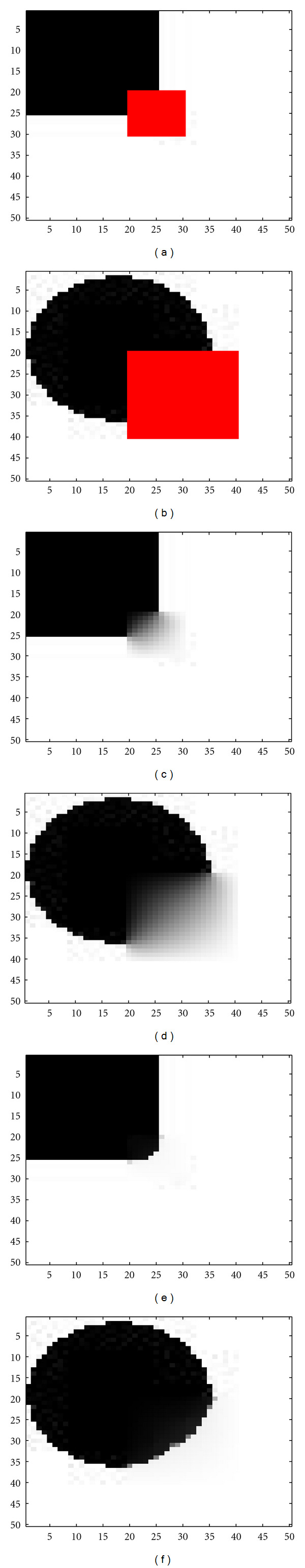
(a) Occluded image, defined by a black square on a white background, the occlusion being represented by a red square; (b) Occluded image, defined by a black circle on a white background, the occlusion being represented by a red square; (c) Inpainted image by diffusion (see (a) for the original degraded image), without any inserted edge in the occlusion; (d) Same as (c) in the circle case; (e) Inpainted image using the missing edges identified by the topological gradient, and then diffusion to fill in the image (see (a) for the original degraded image); (f) Same as (e) in the circle case.

We now illustrate this algorithm on two synthetic examples. We first want to restore a black square, partially hidden by a red square. The degraded image is represented in Figure 4(a).

If no edge is inserted in the hidden zone, then the resolution of a Poisson problem gives a blurred image, as the Laplace operator provides a smooth reconstruction between the black square and the white background, as shown in Figure 4(c). The restored image by the inpainting algorithm is represented in Figure 4(e). Using the edges identified by the topological gradient, the reconstruction by the Laplacian is much better, as there is now an insulating crack between the black and white zones.

The second synthetic example is the reconstruction of a black circle, partially hidden by a red square. The degraded image is represented in Figure 4(b), the restored image by the Laplacian without any inserted edge is shown in Figure 4(d), and the restored image the Laplacian using the edges identified by the topological gradient is represented in Figure 4(f). As one can see on these two synthetic examples, the curvature of the reconstructed edges is continuous in the neighborhood of the boundary of the occlusion. It is not common that an inpainted image has *C*^1^ edges, and for instance, TV-based methods would connect the boundary points with a straight line.

We now explain why we also decided to hybridize the topological gradient and minimal paths methods on a more realistic case.

**Figure 5:**
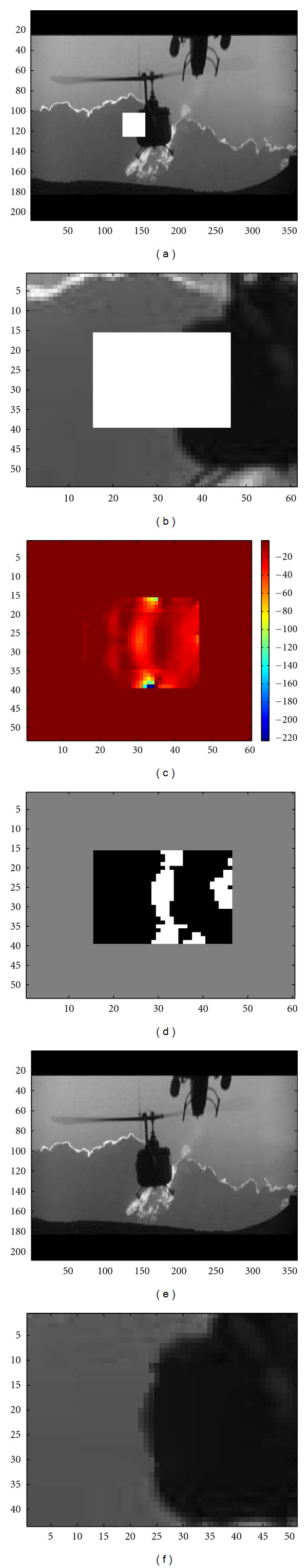
(a) Occluded image (by a white rectangle); (b) Zoom of the occluded zone (see (a)); (c) Topological gradient of (b); (d) Identified edges in the occluded zone by thresholding the topological gradient; (e) Inpainted image using the topological gradient; (f) Zoom of the occluded zone (see (e)).

Figures 5(a) and 5(b) show an example of image, in which we added a mask on a quite large part of the image (*≃*800 pixels). The goal of inpainting is to reconstruct as precisely as possible the original image from the occluded image. We also want the inpainted image to have sharp (unblurred) edges.

Figure 5(c) shows the corresponding topological gradient, provided by the inpainting algorithm. In this case, the topological gradient gives some information about the most probable location of the missing edges. In the inpainting algorithm presented in [25], the idea is then to threshold the topological gradient and to define the edge set of the occluded zone as being the set of points below the threshold. The main issue is that the identified missing edges must be connected in order to avoid blurry effects (due to the Laplacian) in the reconstruction. Then, the threshold is sometimes set manually in order to have connected contours. In our example, the identified edge set is represented by white points in Figure 5(d).

 Figures 5(e) and 5(f) show the corresponding inpainted image. One can see that the reconstruction is not very good, particularly in the top part. This is mainly due to the fact that the missing edges identified by the topological gradient are either connected but thick with a lot of wrong identifications (if the threshold is too small) or discontinuous (otherwise).

The idea is then to apply the fast marching algorithm on the topological gradient obtained during the inpainting process in order to identify connected contours in the hidden part of the image.

## 3. A 2D Algorithm Based on the Minimal Paths and Fast Marching Methods

### 3.1. Minimal Paths

In this section, we describe the standard minimal path technique, adapted to our needs. We refer to [37, 38, 41] for more details about the minimal paths method.

In the following, let *Ω* be the considered image domain. We assume that *Ω* is a regular subset of ℝ^2^. In order to compute some minimal paths, we need to define a potential function, measuring in some sense for any point of *Ω* the cost for a path to contain this point. As we want to identify paths in the topological gradient image, and considering that this potential function must be positive, we will define a potential function as follows:

(21) $$\begin{array}{c} P\left(x\right)=g\left(x\right)-\underset{y\in \Omega }{\min}\left\{g\left(y\right)\right\},\;\;\forall x\in \Omega , \end{array}$$

where *g* is the topological gradient, defined in all the domain *Ω*. We simply shift the topological gradient from its minimal value, in order to obtain a positive function *P*. We can see that the points where the topological gradient *g* reaches its minimal values are quite costless. This is a way to say that these points must be on the minimal paths. On the contrary, if the topological gradient takes high values, then the corresponding potential values lead to very expensive paths.

Once each point has a cost (defined by the potential function), we need to define the corresponding cost of a path. We denote by *C*(*s*) a path, or curve, drawn in the image domain, where *s* represents the arc length. We can now define a functional, measuring the cost of such a path

(22) $$\begin{array}{c} J\left(C\right)=\overset{}{\underset{C}{\int }}\left(P\left(C\left(s\right)\right)+\alpha \right)ds, \end{array}$$

where *α* is a positive real coefficient that represents regularization. The first part of the cost function measures the cost itself of the path *C*(*s*) simply by summing the value of the potential function on this path, and the second part is a regularization term that measures the length of this path. In our applications, *α* is usually very small, as the goal is to connect the most negative parts of the topological gradient, whatever the Euclidean distance is. Note also that we do not consider any regularization terms on the curvature of the contour, as the topological gradient already provides such regularity on the curvature, contrary to TV-based methods. Typically, *α* = 0 would be a good choice, as we really want the minimal path along the topological gradient values, but as the minimum of *P* is 0 (at the minimum of the topological gradient), one has to set *α* to a very small value in order to avoid numerical instability (see (24)).

We now consider a key point *x*_0_ ∈ *Ω* of the image, and *x* will represent any point of the image. The energy *J*(*C*) of a given path *C* can be seen as a distance between the two endings of *C*, weighted by the potential function (and the regularization). The goal is to find the minimal energy integrated along the path *C*. We can now define the weighted distance between key point *x*_0_ and point *x* by

(23) $$\begin{array}{c} D\left(x;x_{0}\right)=\underset{C\in A(x,x_{0})}{\inf}J\left(C\right)=\underset{C\in A(x,x_{0})}{\inf}\overset{}{\underset{C}{\int }}\left(P\left(C\left(s\right)\right)+\alpha \right)ds, \end{array}$$

where *A*(*x*, *x*_0_) is the set of all paths going from point *x*_0_ to point *x* in the image. The idea is that finding the minimal path between points *x* and *x*_0_ is now equivalent to computing the weighted distance function between these two points. If *x* and *x*_0_ are on the same contour of the image, then the minimal path between these two points is obviously a continuous contour of the image, connecting these points. The minimal path has indeed the lowest cost, that is, the points on this path have low topological gradient values. The goal is now to compute the distance function given by (23).

### 3.2. Fast Marching

An efficient way to compute this distance function is to solve a front propagation equation:

(24) $$\begin{array}{c} \frac{\partial ℱ\left(s,t\right)}{\partial t}=\frac{1}{P\left(ℱ\left(s,t\right)\right)+\alpha }\;n_{ℱ}\left(s,t\right), \end{array}$$

where **n**_*ℱ*_(*s*, *t*) is the outer normal unit vector to the front *ℱ*. We initialize the propagation with *ℱ*(*s*, 0), an infinitely small circle centered at key point *x*_0_. This front evolves then with a propagation speed inversely proportional to the potential function. If for example a point in the outer part of the front has a large potential (i.e., a large cost), then the propagation speed will be nearly equal to zero, and the front will not expand much at this point. On the other hand, if the potential is small (i.e., this point is nearly costless), then the propagation speed is large, and the front will quickly propagate in this direction.

The distance *D*(*x*; *x*_0_) introduced in (23), between key point *x*_0_ and point *x*, is then simply the instant *t* at which the front, initialized at key point *x*_0_, reaches point *x*. The algorithm to compute the distance function is called the fast marching technique and is justified by the fact that the distance satisfies the following Eikonal equation:

(25) $$\begin{array}{c} ||\nabla_{x}D\left(x;x_{0}\right)||=P\left(x\right)+\alpha , \end{array}$$

with the initialization *D*(*x*_0_) = 0. We refer to [37, 38, 44, 53, 54] for more details about the fast marching technique and the justification of (25). If *n* is the size of the image, the complexity of this fast marching method is bounded by *𝒪*(*n* · log⁡(*n*)), which is also the complexity of the topological gradient algorithm.

### 3.3. Multiple Minimal Paths

The main issue is now to extend this minimal path technique to more than one keypoint in order to connect several points. This is exactly what we need in order to connect the identified edges by the topological gradient, as we have many identified keypoints (e.g., all negative local minima of the topological gradient) that we want to connect. As explained in [41], the first point of a multiple minimal path algorithm is to reduce the set of keypoints for computational reasons. Moreover, the selected keypoints should not be too close to each other. One usually chooses a total number *N* of keypoints and the first (or main) one. Then, the *N* − 1 other keypoints can be chosen for example as described in [41].

The next step consists of connecting these *N* points. One has to compute the distance function from each of these key points, and the common minimal paths algorithms provide then the Voronoï diagram of the distance and the corresponding saddle points (minimal distance along the edges of the diagram and maximal distance from the keypoints). The Voronoï diagram defines a partition of the image in as many subsets as the number of keypoints. Each subset is defined by the set of points that are closer to the corresponding keypoint than to all others. The saddle points minimize the distance function on the edges of the diagram: minimal distance on the edge and maximal distance to the keypoints [38]. It is useful to compute these saddle points to save computation time, since it reduces the domain of the image where the fast marching computes or updates the weighted distance map.

Finally, the idea is to consider the saddle points as initial conditions for minimizing the distance function. For each saddle point as an initial point, a minimization is performed towards each of the two corresponding keypoints (recall that the saddle points are located at the interface between two subsets of the Voronoï diagram). Each minimization produces a path between the saddle point (initial condition) and a keypoint (local minimum of the distance function). This step is usually called back propagation, as it consists of a gradient descent from the saddle point, back to the linked keypoints. The back-propagation step is straightforward, as there is no local minimum of the distance function, except the keypoints. The union of all these paths gives a continuous path, connecting the keypoints together.

The interesting part of the approach introduced in [41] is that each keypoint should not be connected to all the others, but only to at most two others, as we are looking for a set of closed connected paths. Thus, the keypoints have to be ordered in a way such that they are only connected to the other keypoints that are closest to them in the energy sense [41]. For this reason, we sort all the saddle points from smaller to larger distance, and we first try to connect the pairs of keypoints corresponding to the saddle points of smallest distance. These keypoints are indeed more likely to be connected than distant keypoints, corresponding to saddle points of large potential. Once the close keypoints are connected, we repeat the process with the new closest pairs of keypoints, provided each point remains connected to at most two other ones. At the end of the process, all the keypoints are connected to at most two other keypoints, and the union of all minimal paths between the keypoints represents one (or several) continuous contour of the image. An interesting feature of this method is that the key points are by construction widely distributed around.

If all the selected keypoints are on the same contour of the image, we are almost sure that at the end, they will all be connected together, and we will retrieve the corresponding contour, as the potential function (related to the topological gradient) is very low on this contour. If, on the contrary, one keypoint is not part of the contour, the large values of the topological gradient, and hence of the potential function, will isolate this keypoint from the other ones, and it will not disturb the contour completion process.

### 3.4. Algorithm

The hybrid algorithm we propose is then the following.

**Fast Marching Algorithm Applied to the Topological Gradient**

(i) Compute the topological gradient of the image.
(ii) Set *N* the number of keypoints and choose the *N* keypoints: the main one will be for example the global minimum of the topological gradient, the other ones being the most negative local minima of the topological gradient.
(iii) Compute the distance function (23) with all these keypoints, and the corresponding Voronoï diagram.
(iv) Compute the set of saddle points: on each edge of the Voronoï diagram, determine the point of minimal distance.
(v) Sort all these points of minimal distance, from smaller to larger distance.
(vi) For each of these saddle points, from smaller to larger distance, check if it will not be used to connect two keypoints, one of which is already connected to two other keypoints.
(vii) If this is not the case, perform the back propagation from this point: use this saddle point as an initialization for a descent type algorithm in order to connect the two corresponding keypoints.

It is straightforward to see that this algorithm converges and that at convergence, all the keypoints are connected to at most two other keypoints. This provides one or several continuous contours containing the keypoints. As the first keypoint is usually the global minimum of the topological gradient, it is on one of the main edges of the image. Consequently, using this algorithm, we can identify this edge. Then, it is possible to restart the algorithm, using other keypoints that are not on this identified edge, by initializing, for instance, the first keypoint as the minimum of the topological gradient outside the neighborhood of this edge.

Note that for inpainting applications, the number of keypoints can be set automatically, as the topological gradient takes its minimal values on the edges located on the boundary of the hidden zone, and all these minima (close to the global minimal value of the topological gradient) can be chosen as keypoints.

## 4. Numerical Experiments

### 4.1. Numerical Results for 2D Segmentation

We consider again the grey level image represented in Figure 3(a) for the segmentation application, and we now present the results corresponding to the hybrid method.

**Figure 6:**
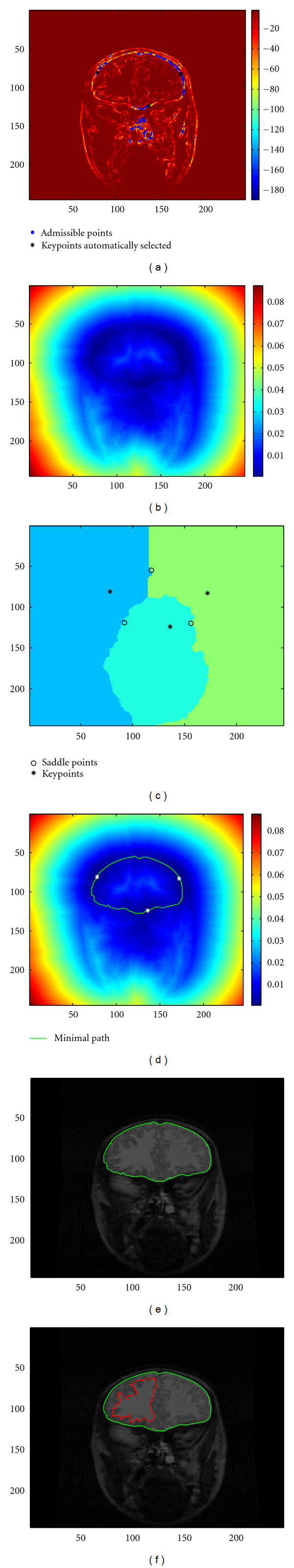
(a) Admissible set of points (i.e., most negative values of the topological gradient) in blue, and 3 keypoints automatically selected in black; (b) Distance function computed from these 3 keypoints with the fast marching algorithm; (c) Corresponding Voronoï diagram, with the 3 keypoints and saddle points; (d) Identified minimal path between the keypoints represented on the distance function; (e) Minimal path between the keypoints represented on the original image; (f) Another identified continuous contour from other keypoints.

Using an automatic thresholding for identifying the most negative values of the topological gradient, Figure 6(a) shows the set of points (or admissible keypoints, in blue), in which we will choose the keypoints for the minimal path algorithm. The first keypoint is set to the minimum of the topological gradient. Then, we have set the number of keypoints to *N* = 3. From the first keypoint, we start the minimal path algorithm, and we choose the second keypoint as being the point (in the admissible set) maximizing the distance to the first keypoint. Then, we start again the minimal path algorithm from these two points, and we set the third keypoint in a similar way. These three keypoints are represented by black points in Figure 6(a). Note that the keypoints can also be (manually) provided by the user, for instance, with the aim of identifying a specific edge of the image.

From these keypoints, we run the minimal path algorithm in order to compute the distance map.  Figure 6(b) shows this distance function. One can clearly see that the distance does not correspond to the Euclidean metric in the plane, as the distance remains very small on the common edge of the 3 keypoints, whereas it takes much larger values outside.

The corresponding Voronoï diagram is represented in Figure 6(c). The three keypoints are still represented by black points. Each color represents the subset *Ω*_*i*_ of points that are closer to keypoint *i* than to the others. For instance, all the points in the green zone are closer to the right keypoint than to any of the two others. This diagram is automatically provided during the distance computation by the fast marching algorithm.

For any *i* ≠ *j*, we consider the interface Γ_*ij*_ = *Ω*_*i*_∩*Ω*_*j*_ between two subsets of the Voronoï diagram. Γ_*ij*_ represents then the set of points equidistant from keypoints *i* and *j*. A saddle point minimizes the distance function on Γ_*ij*_: same distance to keypoints *i* and *j*, minimal distance on Γ_*ij*_. These saddle points are represented by blue points on Figure 6(c). These saddle points can be found during the fast marching propagation as the first meeting points of the fronts starting from each of the keypoints.

From these saddle points, the idea is finally to perform a descent-type algorithm in order to minimize the distance function from the saddle points to the keypoints. We consider a saddle point on an edge Γ_*ij*_ as an initial condition for two minimizations of the distance function, one towards each of the corresponding keypoints (*i* and *j*). Each of these two minimizations provides a continuous path from the saddle point to one of the two keypoints. The union of these two paths connects the two keypoints. This process is done for all pairs of keypoints.

The final set of paths is represented in green on the distance function in Figure 6(d). The three keypoints are also represented (in white). These paths correspond to the contour of the original image that contains the 3 keypoints.

The minimal path is also represented on the original image in Figure 6(e). It also confirms that the identified path perfectly matches the edge we were looking at.

By applying again this algorithm, with other keypoints (selected outside the first identified contour), it is possible to detect other contours of the image.  Figure 6(f) shows, for instance, the first main contour in green and a second one in red. Contrary to the first one, we can see that this contour is not perfectly detected, as the algorithm missed some parts of the contour in the bottom left and top parts of the red zone. One should probably consider more keypoints, and maybe a different regularization coefficient, in order to avoid this phenomenon. But for the application of the topological gradient to image segmentation, the main issue was the discontinuity of the identified contours (see, e.g., [26]). With this approach, we ensure the continuity of the contours, and hence, assuming the edges are well identified, we can obtain a perfectly segmented image.

**Figure 7:**
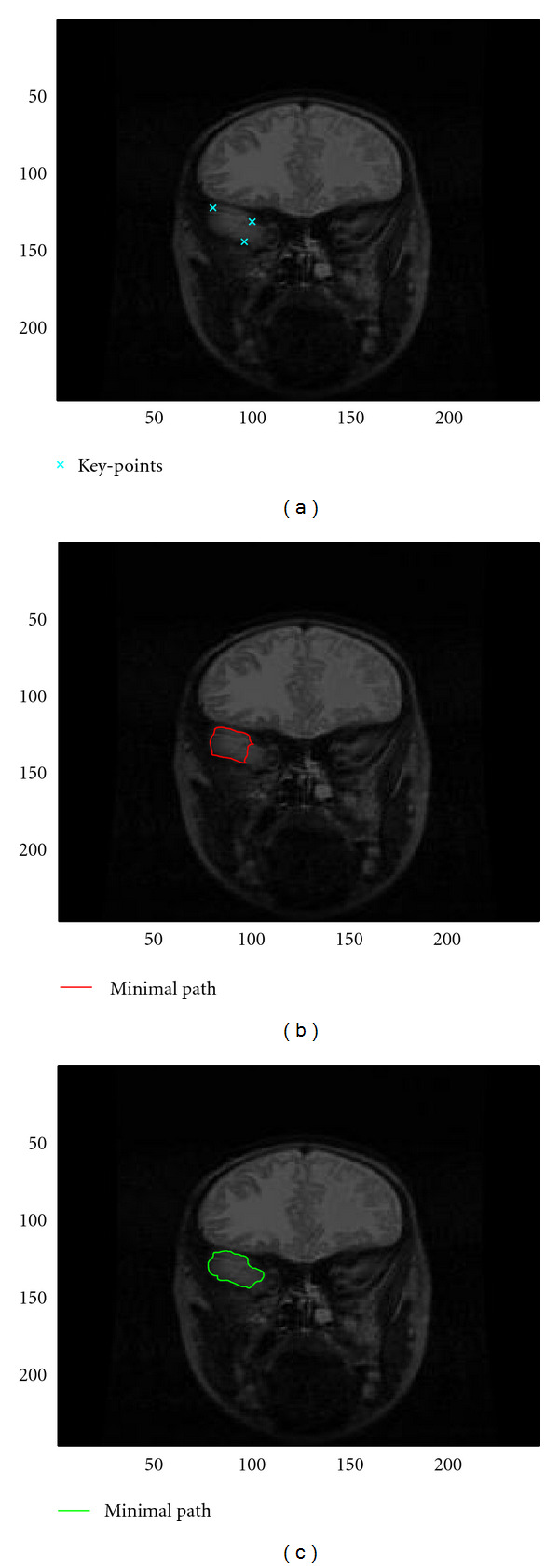
(a) Three selected keypoints on the original image; (b) Contours identified by the fast marching algorithm applied to the standard gradient with the three selected keypoints (see (a)); (c) Contours identified by the fast marching algorithm applied to the topological gradient with the three selected keypoints (see (a)).

Finally, we illustrate the fact that the topological gradient provides better information about the edges of the image than the standard gradient, as previously observed (see Figures 3(b) and 3(c)). We have manually selected 3 keypoints on an edge of the image. These keypoints are represented in blue on Figure 7(a). From these keypoints, we have run the fast marching algorithm (see Section 3.4) applied to both the standard gradient and the topological gradient (hybrid scheme). The identified paths are represented in Figures 7(b) and 7(c), respectively.

The topological gradient clearly provides the best identification of the edge. This can easily be explained by the bad shape of the standard gradient in this region (see Figure 3(b)). On the contrary, the topological gradient is less sensitive to small local variations, and it is more likely to define a potential function than the standard gradient.

### 4.2. Numerical Results for 2D Inpainting

We now consider another application of this hybrid scheme to image inpainting. We recall that the idea of the topological gradient algorithm is to identify the missing edges in the occluded part of the image, and then to reconstruct the image from the solution of a Poisson problem with Neumann boundary conditions [25]. In this application also, it is crucial to have connected contours; otherwise, the reconstruction with the Laplacian will not be satisfactory.

**Figure 8:**
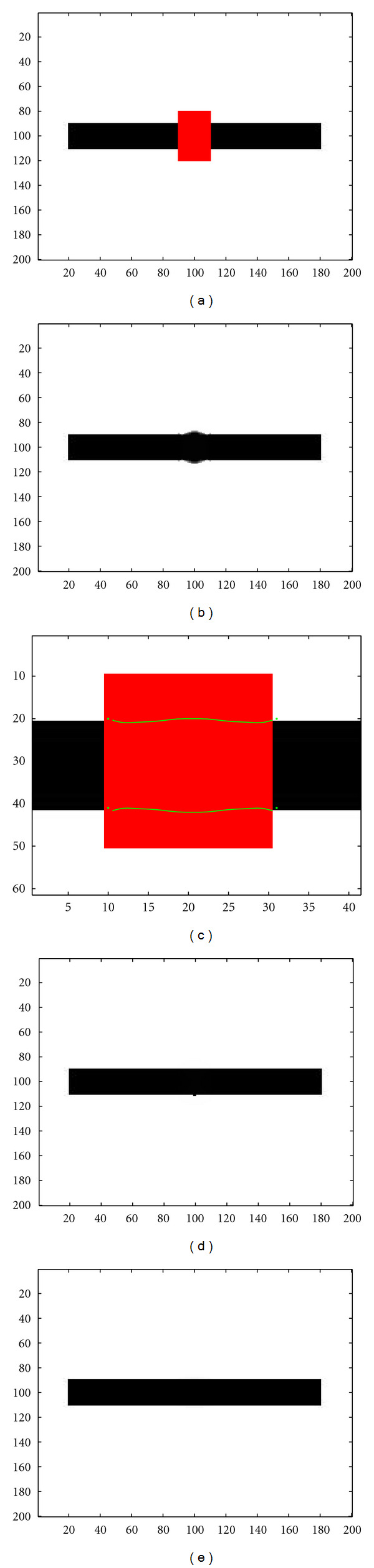
(a) Occluded image (black rectangle) by a red rectangle; (b) Inpainted image using the standard topological gradient; (c) Minimal path between the keypoints represented on the topological gradient; (d) Inpainted image using the hybrid scheme (fast marching algorithm for closing the contours identified by the topological gradient); (e) Inpainted image using a TV-based method.

We first present a comparison between the standard topological gradient approach, a TV-based inpainting method, and the new hybrid scheme. The original image is a black rectangle, and we consider various perturbations of this image.  Figure 8(a) shows a first perturbation of the image, in which the missing region is represented by the red rectangle. The length of the hidden zone is 20 pixels. As previously shown, the missing zone is quite large, and as the identified edges have to be connected in order to avoid blurry effects in the reconstruction, the threshold is set manually to a quite small negative value. And then, the identified edges are then quite thick with a lot of wrong identifications. The reconstructed image by the topological gradient is shown in Figure 8(b). The reconstruction is not very good, as many wrong edges are considered in order to connect the contours.  Figure 8(c) shows the identified minimal path between keypoints (that have been automatically selected, as being the main edges on the boundary of the missing zone) in green, represented on a zoom of the perturbed image.  Figure 8(d) shows the corresponding inpainted image by the hybrid scheme: the image is reconstructed using the topological gradient method, with the edges identified by the minimal path technique. In this case, the reconstruction is perfectly done, and the inpainted image is identical to the original image. A TV-based inpainting method gives the same result (see Figure 8(e)), as the missing zone is not too wide (20 pixels, which is also the size of the black rectangle).

**Figure 9:**
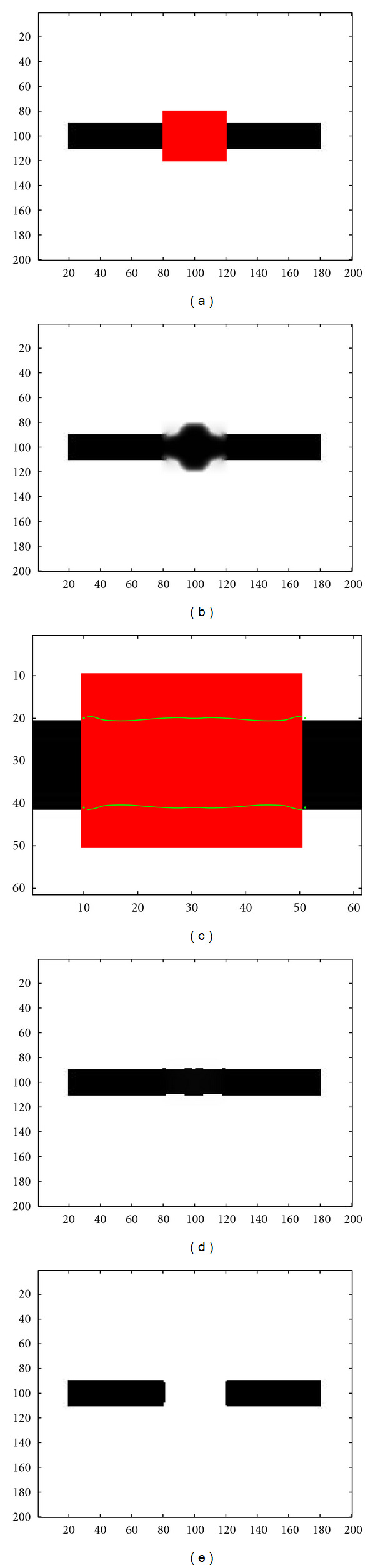
(a) Occluded image (black rectangle) by a red rectangle; (b) Inpainted image using the standard topological gradient; (c) Minimal path between the keypoints represented on the topological gradient; (d) Inpainted image using the hybrid scheme (fast marching algorithm for closing the contours identified by the topological gradient); (e) Inpainted image using a TV-based method.

Figure 9 is similar to Figure 8 in the case of a larger perturbation. The missing zone corresponds now to 40 pixels, twice the size of the black rectangle. In this case, the topological gradient is much less negative near the middle of the hidden zone, and the threshold has to be increased to a smaller negative value in order to have closed contours. The corresponding inpainted image is not good at all. But the minimal path technique still identifies correct edges, and the inpainted image by the hybrid scheme is almost perfect, whereas a TV-based inpainting method does not connect anymore the two regions of the rectangle.

**Figure 10:**
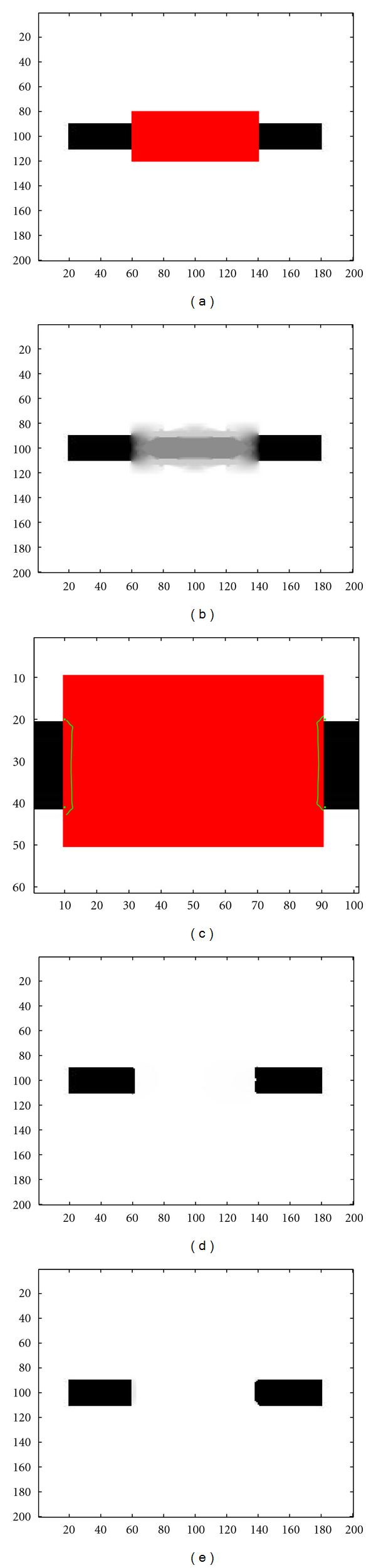
(a) Occluded image (black rectangle) by a red rectangle; (b) Inpainted image using the standard topological gradient; (c) Minimal path between the keypoints represented on the topological gradient; (d) Inpainted image using the hybrid scheme (fast marching algorithm for closing the contours identified by the topological gradient); (e) Inpainted image using a TV-based method.

Figure 10 is similar to Figures 8 and 9, in the case of a larger perturbation. The missing zone now corresponds to 80 pixels, which is much larger than the size of the black rectangle. In this case, the topological gradient still gives unsatisfactory results, due to badly connected edges. Even if the topological gradient has strongly negative values along the missing edges close to the boundary of the perturbation, the missing zone is too wide, and the minimal path technique now connects wrong keypoints, and the inpainted image by the hybrid scheme is no more connected. As before, the TV-based method does not connect the two parts of the rectangle.

We now consider again the occluded image given in Figure 5(a).

**Figure 11:**
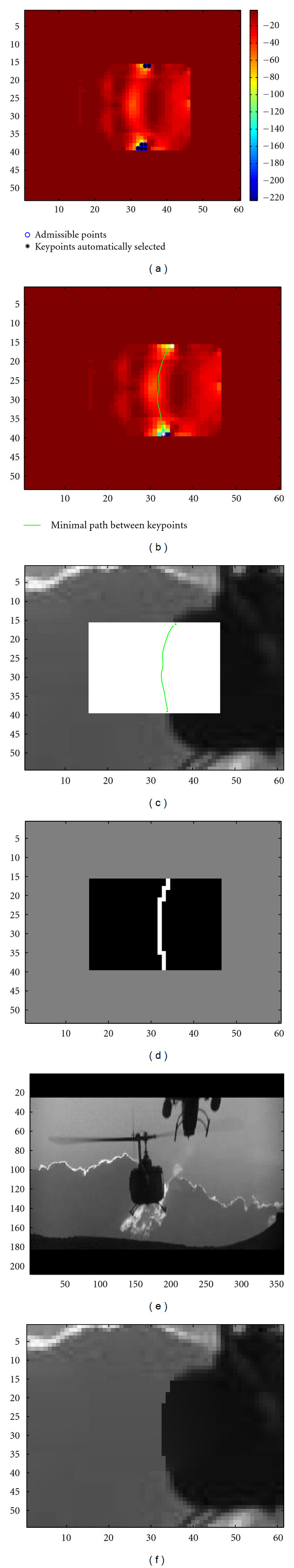
(a) Admissible set of keypoints and selected keypoints on the topological gradient; (b) Minimal path between the keypoints represented on the topological gradient; (c) Minimal path between the keypoints represented on the occluded image; (d) Corresponding identified missing edge in white; (e) Inpainted image using the fast marching algorithm for closing the contours identified by the topological gradient in the hidden part of the image; (f) Zoom of (e).

After thresholding the topological gradient, several points (identified by blue circles) have been identified and define the admissible set of keypoints represented in Figure 11(a). We choose then the most negative point of the topological gradient as the first keypoint and then the further admissible point as the second one. The keypoints are represented by a large black point on the same image. They are located on the edge of the domain, as the inpainting topological gradient always takes its minimal values there.

Then, the minimal path algorithm is run, and it provides a path between the keypoints, represented in green in Figure 11(b). We can see that the path follows very well the valley line of the topological gradient, from one side to the other. By choosing 3 keypoints instead of 2, there will be another keypoint on the bottom edge, near the first one, and it will simply add a small contour located all along on the edge of the domain, and consequently, there is absolutely no impact on the reconstruction of the hidden part of the image.

Figure 11(c) shows the same identified path represented on the occluded image. This allows one to see that the path clearly gives a good approximation of the missing edge and also that the topological gradient is very powerful for this identification problem. The corresponding identified edge set is represented in Figure 11(d). This image should be compared with the thresholded edge set of Figure 5(d). From these two images, we can conclude that the minimal path algorithm is an excellent tool for extracting the valley lines of the topological gradient.

Finally, using this minimal path as the set of missing edges in the occluded zone, the inpainting topological gradient algorithm produces a much better reconstructed image, shown in Figures 11(e) and 11(f). The quality of the image is very good, as the missing edges used for the reconstruction are connected, and the Laplace operator will not produce any blurring effect due to a discontinuous contour. Note that there are some small discontinuities on the top left boundary due to the fact that we used the Neumann solution of the perturbed problem. The construction of a Dirichlet solution would be better, but it is also much more difficult to solve the Dirichlet problem in this case, as it is ill posed. This example confirms that the quality of all topological gradient applications in image processing can be improved by replacing a simple thresholding technique by a minimal path algorithm.

As already shown in [25], the topological gradient extrapolates the edges and their curvature in the missing part of the image (see also Figure 11(d), in which the identified edge is not a straight line), contrary to total variation-based methods. Thus, provided the identified missing edges are connected (this point is now ensured by the application of the fast marching algorithm to the topological gradient), the inpainted image has edges with continuous curvature, which is not the case with many other inpainting schemes.

## 5. Conclusions and Perspectives

We have introduced a hybrid scheme, based on one side on the topological gradient for edge detection, and on the other side on the fast marching and minimal paths methods for contour completion. These approaches allow us to extract connected contours in 2D images and to solve the main issue of all topological gradient-based algorithms for image processing problems (discontinuity of the edges). Moreover, the minimal path algorithm does not degrade the complexity of the topological asymptotic analysis.

We have considered two specific applications in image processing: segmentation and inpainting. In the first one (segmentation), we showed that the topological gradient is more efficient than the standard gradient for edge detection and the hybrid scheme provides better results than the fast marching method applied to the standard gradient of the image. In the second application (inpainting), we showed that the hybrid scheme particularly improves the quality of the inpainted image, as the contour completion ensures a nonblurred inpainted image and as it also helps removing the manual thresholding of the topological gradient.

The hybrid scheme is very efficient and quite automatic, as there is no more thresholding process. The topological gradient algorithm has been shown in previous inpainting articles to propagate the main edges inside the hidden zone, with some continuity of their curvature, and the use of a minimal path technique helps detect the valley lines of the topological gradient. The main drawback of the hybrid scheme is the same as for the standard topological gradient algorithm: the image is filled in with the Laplacian, and this part has to be improved in order to also recover texture information. Some preliminary results show that it is possible with the same kind of approach, thanks to higher order operators.

An interesting and natural perspective is to apply this hybrid scheme to 3D images and movies. The topological gradient can very easily be extended to 3D images. The minimal path technique has also been adapted to the identification of tubular structures in 3D [37]. Another perspective consists of dealing with the changes of topology of the edges in order to automatically detect bifurcations and T-junctions.
